# Context-Aware Beam Selection for IRS-Assisted mmWave V2I Communications

**DOI:** 10.3390/s25133924

**Published:** 2025-06-24

**Authors:** Ricardo Suarez del Valle, Abdulkadir Kose, Haeyoung Lee

**Affiliations:** 1 Department of Electronic Engineering, University of Surrey, Guildford GU2 7XH, UK; ricardo.su.dv@gmail.com; 2 Department of Computer Engineering, Abdullah Gül University, Kayseri 38080, Türkiye; 3 School of Physics, Engineering and Computer Science, University of Hertfordshire, Hatfield AL10 9AB, UK; h.lee@herts.ac.uk

**Keywords:** mmWave, V2X, RIS, machine learning, multi-armed bandit

## Abstract

Millimeter wave (mmWave) technology, with its ultra-high bandwidth and low latency, holds significant promise for vehicle-to-everything (V2X) communications. However, it faces challenges such as high propagation losses and limited coverage in dense urban vehicular environments. Intelligent Reflecting Surfaces (IRSs) help address these issues by enhancing mmWave signal paths around obstacles, thereby maintaining reliable communication. This paper introduces a novel Contextual Multi-Armed Bandit (C-MAB) algorithm designed to dynamically adapt beam and IRS selections based on real-time environmental context. Simulation results demonstrate that the proposed C-MAB approach significantly improves link stability, doubling average beam sojourn times compared to traditional SNR-based strategies and standard MAB methods, and achieving gains of up to four times the performance in scenarios with IRS assistance. This approach enables optimized resource allocation and significantly improves coverage, data rate, and resource utilization compared to conventional methods.

## 1. Introduction

The advent of millimeter-wave (mmWave) technology has sparked a transformative wave in next-generation wireless communication. In this dynamic landscape, Vehicle-to-Everything (V2X) communication emerges as a pivotal force poised to reshape the future of transportation systems [1]. By enabling seamless interaction among vehicles, infrastructure, pedestrians, and other road users, V2X holds the promise of fostering safer, more efficient, and smarter transportation networks [2]. However, leveraging mmWave for V2X introduces significant challenges due to the high-frequency nature of mmWave signals, including increased propagation loss, susceptibility to interference, and mobility constraints [3]. Additionally, mmWave small cells operate with highly directional narrow beams to maintain high data rates, but this results in frequent beam switching and handovers as vehicles rapidly move between beam sectors, adding further complexity to maintaining stable and reliable communication.

To fully harness mmWave’s potential for V2X communication, researchers and engineers are delving into innovative solutions aimed at enhancing signal propagation, coverage, and reliability. One particularly promising avenue gaining momentum is the integration of Intelligent Reflecting Surfaces (IRSs) into mmWave V2X systems [4]. Outfitted with passive reflecting elements, these surfaces excel at redirecting signals when a direct link between User Equipment (UE) and a mmWave Base Station (BS) is obstructed [5]. IRS technology emerges as a focal point in the research community, holding the potential to significantly boost spectral efficiency and expand communication range [6].

The efficacy of IRS-assisted mmWave V2X communication hinges not only on deploying IRSs but also on tailoring their functionality to the specific demands and dynamics of the mmWave V2X environment [7]. Context-awareness becomes paramount in this context, enabling the system to dynamically respond to real-time changes in vehicular mobility, traffic patterns, and environmental conditions [8]. Employing context-aware beam selection strategies alongside IRS technology proves instrumental in enhancing communication quality and coverage, minimizing latency, and ensuring the reliability of mmWave V2X communication across diverse scenarios [9].

## 2. Related Works

Beam management and selection, a fundamental aspect of IRS-assisted mmWave V2X systems, have been at the forefront of investigation. However, efficiently managing the beams in such systems, especially coordinating between the BS and IRS for two-hop transmission, is challenging. Several papers have contributed novel approaches to this domain, enhancing the efficiency of mmWave V2X communication. A comprehensive survey by [10] offers a broad overview of IRS applications in vehicular communications, both ground-based and aerial, highlighting the potential and challenges of IRS-aided 6G vehicular communications. Additionally, ref. [11] discusses various use case scenarios for IRS-enabled V2X communications, particularly focusing on vehicular edge computing and IRS-enabled drone communications to minimize vehicle computational time.

A machine learning-based approach proposed in [12] includes two phases: situational and mobility awareness. In the situational awareness phase, a model at the BS predicts the optimal IRS setup for a UE’s location. The mobility awareness phase, at the UE, predicts UE mobility. This allows for predicting the optimal IRS configuration, and significantly reduces system overhead during initial access. In highly dynamic scenarios, it achieves higher spectral efficiency by using predicted UE locations. Leveraging location information, ref. [13] examines the impact of prior location data on channel parameter estimation in IRS-aided mmWave MIMO (Multiple-Input Multiple-Output) systems. It presents directional training beams based on rough location data and employs atomic norm minimization for channel parameter extraction. The authors of [14] introduce real-time software-controlled IRSs and examine their optimal placement in wireless networks, addressing reliability requirements for V2X communications. Furthermore, ref. [15] introduces a deep reinforcement learning algorithm for beamforming optimization in mmWave vehicle-to-infrastructure (V2I) communication systems. This novel algorithm tackles the non-convex and time-varying nature of joint beamforming matrix optimization for the BS and the IRS. The results demonstrate substantial network performance improvement, especially in dense V2I network scenarios.

One approach, as proposed in [16], introduces multi-beam training methods for IRS-assisted multiuser communication, designed to reduce training time without compromising passive beamforming performance. It achieves this by partitioning IRS elements into sub-arrays and implementing a multi-beam codebook, thus enabling efficient beam direction control for different users. Ref. [17] explores the use of IRSs in a millimeter-wave vehicular communication network, introducing schemes to reduce channel estimation overhead and considering rate maximization in the uplink. Lastly, the study in [18] employs IRSs to mitigate Inter-Cell Interference (ICI) by using graph theory to minimize beam collisions.

### Our Contributions

These research findings collectively underscore the evolving landscape of IRS-assisted mmWave V2X communication, emphasizing innovation in beam management and selection as a key enabler for its widespread adoption and the improvement in next-generation vehicular communication systems. In addition, these traditional beam selection strategies in vehicular mmWave networks often rely on instantaneous signal strength or static location mapping, which do not reflect future link duration or vehicle mobility patterns. As a result, they tend to cause frequent handovers and unstable links. In contrast, our proposed context-aware framework predicts longer-lasting beam assignments based on high-level contextual factors, reducing switching events and improving average link duration. In this paper, we proposea Context-aware Multi-Arm Bandit (C-MAB)-based beam selection for IRS-assisted vehicular communication by incorporating vehicle mobility context under a realistic vehicular mobility environment. The aim is to establish long-lasting connections, unlike signal strength-based approaches for end-to-end V2I communication. Note that, unlike standard C-MAB models, our approach considers a spatiotemporal environment where vehicle mobility and shared beam resources create interaction between decisions. While most C-MABs assume static user contexts and independent arm outcomes, our setting involves overlapping beam coverage, prioritized scheduling, and varying mobility patterns. Therefore, we define reward as the total connection time achieved under a beam, conditioned on the vehicle’s context and future trajectory. This formulation aligns more closely with real-world beam stability concerns and differentiates our work from typical bandit designs that focus only on immediate signal gain or throughput. The following describes the main contributions of this paper.

- *Context-driven beam and IRS selection*: We introduce a novel approach that leverages a range of contextual factors to enhance the beam and IRS selection process. By considering variables such as vehicle mobility information (e.g., travel directions and relative locations) and traffic light information, we improve the beam and IRS selection process to enable longer-lasting connections. This context-aware selection approach not only improves the overall connectivity quality but also extends the connection time, a critical factor in V2X communication, especially for mission-critical applications.
- *Predictive testing tool for IRS locations*: Recognizing the importance of IRS placement in achieving optimal signal reflections, we offer a practical tool for assessing the effectiveness of IRS locations. This tool empowers network planners and engineers to simulate IRS deployments and assess their impact on signal propagation before actual implementation. By doing so, we mitigate the risk of suboptimal IRS placement, thereby enhancing the overall performance and robustness of mmWave V2X communication systems.
- *Utilization of traffic light waiting times*: This is the first work to incorporate waiting times at traffic lights as contextual data for predicting beam sojourn times in IRS-assisted V2I communication. This predictive insight allows for the development of proactive mobility and resource management strategies. By factoring in traffic light cycles, we can optimize beam switching, resource allocation, and mobility planning, ensuring that the V2I communication system remains seamless and uninterrupted, even in stop-and-go traffic scenarios. Note that the traffic light status can be acquired through standard V2X interfaces supported by modern infrastructure. Traffic controllers broadcast phase and timing information (e.g., current light status, remaining time, next transition) using SPaT messages [19]. These data can be received directly by the base station via wired connectivity or wirelessly via Roadside Units (RSUs) that act as V2I relays. In addition, vehicles can share their perception of the traffic light status through Cooperative Awareness Messages (CAMs) or Basic Safety Messages (BSMs), which include location, speed, heading, and, in some cases, braking events that suggest deceleration due to traffic light stops. These standardized messages allow the base station to build a synchronized and real-time environmental context, which includes the dynamic state of the traffic signal affecting each vehicle’s route.

In summary, our contributions encompass not only context-aware beam and IRS selection for prolonged connection times but also a practical testing tool to optimize IRS deployment strategies. Moreover, by harnessing waiting times at traffic lights as predictive context, we enable the development of proactive strategies to enhance mobility management and resource allocation in mmWave V2X communication. These innovations collectively pave the way for more robust, reliable, and efficient vehicular communication systems, crucial for the advancement of smart and safe transportation networks. The remainder of this paper is organized as follows: Section 2 describes the considered scenario and formulation of the beam selection problem. In Section 3, the C-MAB based beam selection algorithm is elaborated. The experimental results are explained in Section 4 to show the effectiveness of our proposed algorithm. Finally, we draw important conclusions in Section 5.

## 3. Scenario Setup and Problem Formulation

We examine a mmWave small cell designed to improve throughput performance and network capacity for downlink transmission. The small cell BS under consideration is equipped with an antenna array directed along predefined orientations, though the number of radio frequency (RF) chains is fewer than the number of antennas. Each antenna may consist of multiple elements that form a beam directed towards a specific region. As described in [20], the coverage area of a beam, or beam sector, is ideally spatially separated. Given the limited RF chains, only a subset of antennas can be activated for downlink at any time.

**Figure 1:**
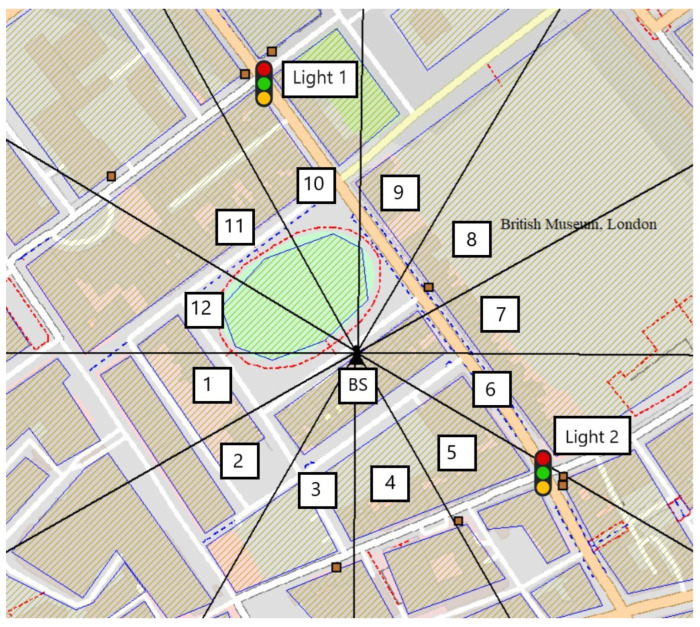
A scenario of a network which consists of a mmBS with 12 beam sectors and multiple IRS nodes shown on a real-world map of around Bedford Square in London.

The scenario is depicted in Figure 1. The mmWave base station (mmBS), located in central London, UK, is equipped with 12 antennas oriented to ensure broad coverage. Due to a limited number of RF chains, the mmBS can activate up to four beams simultaneously, supporting a maximum of four vehicles at once. Beam coverage is determined by the vehicle’s presence within a beam’s geometric footprint and the achieved SNR meeting the threshold for link activation. We adopt a two-slope path loss model to distinguish line-of-sight (LOS) and non-line-of-sight (NLOS) propagation. If the estimated SNR falls below a threshold (e.g., 2 dB), coverage is not considered valid. The data rate is modeled as a constant value during beam association, consistent with baseline mmWave assumptions and to isolate the impact of connection time from PHY-layer variations. Resource utilization reflects the number of active beams simultaneously serving distinct vehicles, constrained by beamforming hardware limits (i.e., max four beams). A higher beam sojourn time implies better utilization per beam, as it reduces frequent reconfigurations and idle slots.

To further enhance signal coverage and alleviate potential blockages in the urban environment, several IRS nodes are strategically deployed within the area. These IRS nodes dynamically reconfigure signal propagation paths, improving beam delivery in NLOS conditions. Note that the illustrated sector areas are approximate; actual coverage depends on the channel model, antenna configuration, and IRS-assisted reflections.

This work assumes a carrier frequency of 28 GHz, which has been widely adopted in 5G NR-based mmWave V2X studies [21,22,23]. While traditional DSRC (Dedicated Short-Range Communications) and C-V2X (Cellular V2X) systems operate in the 5.9 GHz band, the 3GPP n257 (26.5–29.5 GHz) is considered a promising band for ultra-high data rate and low-latency vehicular applications, particularly in urban environments with managed infrastructure and directional communication. The adjacent 26 GHz band (n258) is already approved for V2X use in Europe [22]. Field trials such as autonomous beam-steered measurement campaigns [23] further validate the viability of the 28 GHz bands for vehicular communications. Thus, we consider 28 GHz mmWave communication with its path loss model [21] expressed as:

(1) $$PL\left(d\right)=PL\left(d_{0}\right)+10n\log_{10}\left(d/d_{0}\right)+X_{g}.$$

Here, *d* refers to the distance between the mmBS and vehicle antennas in meters while $X_{g}$ represents the channel fading (excluded in our analysis), and $PL(d_{0})$ denotes the free space path loss (FSPL) in decibels (dBs). The FSPL depends on the carrier frequency $f_{c}$, and is given by $10\log_{10}\left({\left(4\pi d_{0}f_{c}/c\right)}^{2}\right)$, with $d_{0}=1\;m$. We assume a 5 m height difference between the BS and the vehicle, such that the 2D distance $\hat{d}$ between two points is related to *d* as $d=\sqrt{{\hat{d}}^{2}+5^{2}}$.

Assuming that vehicles have steerable beam antennas capable of tracking the BS, the signal-to-noise ratio (SNR) for a vehicle being served is given by:

(2) $$SNR=p_{0}-PL\left(d\right)+G_{tx}+G_{rx}-N,$$

where $p_{0}$ represents the reference power which is the received power at a reference distance of $d_{0}=1\;m$. $G_{tx}$ and $G_{rx}$ are the transmitter and receiver antenna gains, respectively, *N* is the noise, including thermal noise and the receiver noise figure.

As aforementioned, the deployment of mmWave small cells for V2X communication introduces a distinct challenge, as narrow beams are used. The short time a vehicle is present within a beam’s coverage results in frequent handovers and increased signal overhead. In addition, due to the fluctuating nature of the channel caused by vehicle mobility, a more conservative approach-using a fixed, robust modulation scheme may be more suitable than an adaptive modulation scheme. Thus, we assume the BS uses a constant, resilient modulation scheme, regardless of the reported SNR. To maximize data transmission in downlink, the radio resource allocation strategy should prioritize vehicles that stay within the beam for the longest periods.

Let *R* denote the fixed data rate assigned to each beam when serving a vehicle within its beam sector. Vehicles that leave the beam sector experience a zero data rate. Consider a set B(t) of available beams from the mmWave small cell BS at time *t*, where |B(t)| can be at most 4, representing the maximum number of simultaneously active beams. Let $R_{i}\left(t\right)$ denote the transmission rate of beam *i* at time *t*, where $R_{i}\left(t\right)=R$ if a vehicle is being served, and $R_{i}\left(t\right)=0$ during handovers, when waiting data from the macro BS, or when no vehicle is within the beam’s coverage. The objective of the proposed resource allocation strategy is to maximize the total data transmission across all beams over a given time period *T*. Minimizing handovers is crucial to achieving this goal, as each handover introduces a data transmission interruption. Let $S_{i}$ represent the number of serving vehicle switches for beam *i* during *T* and let $T_{ij}$ denote the sojourn time of *j*th vehicle served by beam *i*. By maximizing the vehicle sojourn time within a beam sector, the frequency of handovers is reduced, thereby increasing overall transmission time. Consequently, the resource allocation problem can be formulated as:

(3) $$\max\left(\sum_{i\in B(t)}\int_{0}^{T}R_{i}\left(t\right)dt\right)\;\mathrm{or}\;\max\left(\sum_{i\in B(t)}\sum_{j=1}^{S_{i}}T_{ij}\right).$$

To maximize data transmission, the mmBS utilizes all available beams. When a vehicle receives service within a beam sector, the corresponding beam becomes unavailable for other vehicles, and the BS selects another beam from the available set to serve a new vehicle. The BS can either select a vehicle at random or prioritize the vehicle with the highest SNR. However, as we will demonstrate, the conventional strategy of selecting the vehicle with the highest SNR may not be optimal for mmWave vehicular networks due to the narrow beamwidth and frequent handovers. Given the restricted mobility of vehicles within urban street layouts, we propose leveraging vehicle mobility information as an indirect indicator of the street’s topology. By constructing vehicle profiles based on their mobility patterns, this contextual information can be utilized for beam selection. Mobility data, such as vehicle orientation, position, and distance from the BS (obtained through local measurements or timing advance), can be employed to enhance the efficiency of beam assignment.

## 4. Proposed Contextual Multi-Armed Bandit Learning Design

In our previous work [24,25], we demonstrated the effectiveness of the Contextual Multi-Armed Bandit (C-MAB) algorithm in optimizing beam-level communication in mmWave vehicular networks. Based on this, our subsequent study [26] extended C-MAB to relay-assisted V2V-based V2X communication strategies, emphasizing the importance of contextual information in dynamic environments. These studies highlighted how factors such as road layout and blockages significantly impact connectivity, which requires careful selection of contextual data to improve decision making [24,26]. In this paper, we study a new environment by incorporating IRSs into vehicular communication, addressing the challenges posed by dynamic blockages and harsh propagation conditions in mmWave networks. Unlike relays, IRS extends coverage passively and efficiently but faces key challenges: it lacks signal amplification, limiting effectiveness in weak links; requires continuous phase shift adaptation to vehicle mobility; and demands precise beam alignment, making real-time coordination complex. Additionally, IRS placement is constrained by existing infrastructure, requiring strategic positioning for optimal coverage. To tackle these challenges, we incorporate diverse contextual factors—such as vehicle travel direction, mmBS location, and traffic light information—into contextual learning algorithm for optimized beam selection, ensuring stable and prolonged connections. Our approach strategically leverages IRSs to enhance vehicular communication while mitigating their limitations, enabling robust and adaptive connectivity in dynamic urban environments. The following seven contexts are incorporated into the proposed C-MAB framework to enhance beam selection and connectivity:

- Vehicle Travel Direction: {North, East, South, West};
- Vehicle Travel Direction with respect to mmBS: {Towards, Away};
- Vehicle distance to mmBS: {Close, Mid, Far};
- Traffic light status: {Red, Green};
- Remaining time for traffic light color change: {Small, Mid, Big};
- Vehicle Travel Direction with respect to traffic light: {Towards, Away};
- Vehicle distance to traffic light: {Close, Mid, Far}.

The algorithm utilizes seven distinct contextual features, each represented by a single character. These characters are concatenated into a seven-character string that encodes the complete context. Each character corresponds to one specific context, capturing its current discrete state. For instance, a context string “SFTRSFT” represents a vehicle that is traveling South (S), is Far (F) from the mmBS, and moving Towards (T) it; while the nearest traffic light is Red (R), has a Small (S) remaining time before switching, and the vehicle is Far (F) and moving Towards (T) the light. This context representation balances environmental richness with computational efficiency, enabling intelligent and adaptive beam–IRS decision-making in highly dynamic vehicular environments.

**Figure 2:**
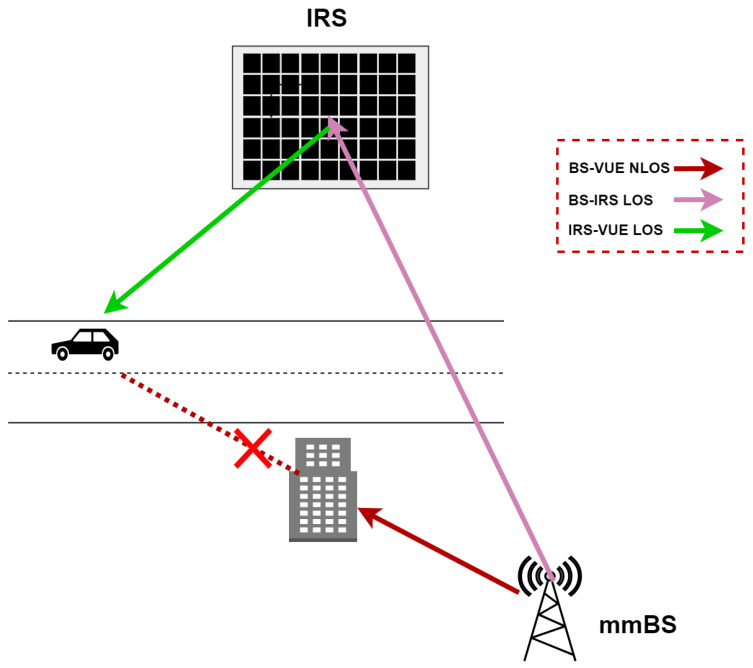
IRS-assisted beam communication under blockages. The dotted red line with a cross symbol represents a blocked (NLOS) path between the mmBS and the vehicle UE.

The proposed C-MAB algorithm is designed to integrate IRS-assisted links, ensuring improved coverage and seamless beam allocation in obstructed environments. To mitigate blockages, IRSs are employed to extend coverage to vehicles experiencing connectivity disruptions, leveraging V2I communication when needed, as illustrated in Figure 2. By intelligently selecting direct or IRS-assisted transmission, the algorithm enhances overall network reliability and connectivity in dense urban settings. It is important to note that, unlike active antenna arrays or smart antennas, IRS units do not require RF chains or power-hungry circuitry, which makes them highly energy-efficient and cost-effective. However, this also introduces certain limitations. First, since IRS elements only reflect incoming signals, they depend heavily on favorable deployment geometry—that is, they must be positioned where they can effectively “see” both the BS and the vehicle. In addition, due to path loss from two-hop reflection (BS→IRS→UE), IRS-assisted links often require stronger incident signals or tighter phase alignment to be beneficial compared to direct line-of-sight links. In this work, we adopt the widely used assumption of quasi-static phase configurations for the IRS, consistent with prior works [27]. The IRS is pre-configured to reflect signals toward designated zones based on its physical orientation and deployment geometry. The current algorithm does not perform real-time phase shift optimization but rather selects among available beam–IRS combinations based on learned context-specific rewards. This abstraction allows us to focus on the high-level contextual beam selection problem without entangling it with the physical-layer phase design. However, in practice, the integration of dynamic IRS control—particularly under high-mobility vehicular scenarios—is an open challenge and represents a valuable direction for future work.

Algorithm 1 presents the C-MAB algorithm designed for a communication scenario where a BS with with *B* beams and *S* distributed IRSs serve vehicles in a dynamic environment. The objective is to optimize beam and IRS selection by leveraging contextual information (*c*), as detailed in this section, to enhance decision-making. To give an example of operation of the C-MAB algorithm, let C represent the set encompassing all possible contextual information, and 〈b,s,c〉 denote an ordered triple consisting of beam *b*, IRS *s*, and context *c*. The system records rewards for all combinations of beams, IRSs, and contexts, forming the set $Q_{C}=\left\{q_{\langle b,s,c\rangle }|b\in B,s\in S,c\in C\right\}$. When a beam $b_{i}$ and an IRS $s_{i}$ are selected for transmission at time *t*, an instantaneous context $c_{i}\left(t\right)$ is observed. Subsequently, after a vehicle service duration of Δt, the reward $q_{i}\left(t+\Delta t\right)$ associated with the current context is measured. This reward is then utilized to update the average reward value of context $c_{i}\left(t\right)$ using the formula:

(4) $${\bar{q}}_{c_{i}}\left(t+\Delta t\right)=\frac{{\bar{q}}_{c_{i}}\left(t\right)\cdot n_{c_{i}}\left(t\right)+q_{i}\left(t+\Delta t\right)}{n_{c_{i}}\left(t\right)+1},$$

where $n_{c_{i}}\left(t\right)$ represents the number of times the current context has been observed in the past.

The learning strategy adopts an exploration-first approach. The algorithm initializes parameters, including expected rewards ($Q_{b}^{s}\left(c\right)$) and observation counts ($N_{b}^{s}\left(c\right)$), for all beam–IRS configurations. In the exploration phase, a subset of beams is activated randomly, and vehicles are also selected randomly based on observed contexts. Depending on beam availability, transmission occurs directly or via an IRS. The system updates expected rewards and observation counts through a weighted update rule to adapt to changing conditions. Transitioning to the exploitation phase after a specified number of exploration rounds, the algorithm intelligently selects the top *K* beams with the highest expected rewards. For each active beam, the algorithm determines the optimal transmission mode (direct or IRS-assisted) to maximize rewards. Upon connection loss due to blockage or vehicle movement, the reward values for each context–beam–IRS triplet are updated to optimize long-term performance.
**Algorithm 1** Contextual MAB for Beam and IRS Selection1:**Input:** Number of beams *B*, Number of IRSs *S*, Contexts *C*, Exploration rounds $T_{e}$, Maximum active beams *K*, Counter for observed (beam, context) pair *N*2:**Initialize:** $Q_{b}^{s}\left(c\right)\leftarrow 0$ and $N_{b}^{s}\left(c\right)\leftarrow 0$ for all b∈[1,B], s∈[1,S], and c∈C3:**procedure** Exploration4:      **while** $t\leq T_{e}$ **do**5:           Observe context $c_{t}$6:           Randomly activate a set of beams $K_{t}$ from *B*7:           Randomly select vehicle $v_{t}$ based on $c_{t}$8:           **if** There exists $b_{t}\in K_{t}$ such that Beam $b_{t}$ can reach $v_{t}$ **then**9:                Transmit directly via Beam $b_{t}$10:              Record reward $r_{t}$11:         **else**12:              Joint transmission via IRS13:              Record reward $r_{t}$14:         **end if**15:         Update $N_{b_{t}}^{s_{t}}\left(c_{t}\right)\leftarrow N_{b_{t}}^{s_{t}}\left(c_{t}\right)+1$16:         Update $Q_{b_{t}}^{s_{t}}\left(c_{t}\right)\leftarrow Q_{b_{t}}^{s_{t}}\left(c_{t}\right)+\frac{1}{N_{b_{t}}^{s_{t}}\left(c_{t}\right)}\cdot r_{t}$17:    **end while**18:**end procedure**19:**procedure** Exploitation20:     **while** $t>T_{e}$ **do**21:         Observe context $c_{t}$22:         Select a beam **b** with highest $Q_{b}^{s}\left(c_{t}\right)$ values for active transmission23:         **for** Active Beam $b_{t}$ **do**24:              **if** Beam $b_{t}$ can reach $v_{t}$ **then**25:                   Transmit directly via Beam $b_{t}$26:                   Record reward $r_{t}$27:             **else**28:                   Joint transmission via IRS29:                   Record reward $r_{t}$30:            **end if**31:            Update $N_{b_{t}}^{s_{t}}\left(c_{t}\right)\leftarrow N_{b_{t}}^{s_{t}}\left(c_{t}\right)+1$32:            Update $Q_{b_{t}}^{s_{t}}\left(c_{t}\right)\leftarrow Q_{b_{t}}^{s_{t}}\left(c_{t}\right)+\frac{1}{N_{b_{t}}^{s_{t}}\left(c_{t}\right)}\cdot r_{t}$33:        **end for**34:    **end while**35:**end procedure**

## 5. Results and Discussion

In the section, we present experimental results for the proposed C-MAB machine learning based beam selection. The scenario considered for experiment is presented in Figure 1, with the system parameters outlined in Table 1. In this scenario, we consider a standalone single mmBS with 12 beams. At any given time, the BS can only activate four beams for service. Vehicles are created and absorbed at certain locations on the map. Those locations are the main entrance point to the city and the exit point from the city. Moreover, we also included several main parking spots in the city as vehicle creation and absorption locations. A pair of vehicle creation and absorption locations is used to create a route simulating a vehicle either passing through the city, entering to or exiting from the city. We use A-STAR path finding algorithm [28] to establish the route for vehicles. We assume that each of these vehicles requires downlink data service when entering the small cell. In our simulation, there are 50 vehicles which continuously travel on the map. We simulate three hours of operation, during which the base station begins with full exploration for learning, and then it switches to full exploitation after the first 50% of the simulation time. As the Explore-First strategy stops triggering exploration after the learning phase, this enables us to focus on the study of learning effectiveness acquired during the learning phase. The key simulation parameters used in our experiments are summarized in Table 1. Experiments are conducted via our own developed Python Mobility Simulation Platform (PyMoSim, version 0.8.10). The detailed documentation of PyMoSim, along with example usage scenarios, is publicly accessible at https://github.com/cfoh/pymosim-doc, accessed on 22 June 2025. We plan to release the full source code of PyMoSim and our scenario setup code in the near future).

The proposed C-MAB algorithm is selected for its lightweight, model-free nature, which makes it well-suited for real-time decision-making at the network edge. Unlike deep reinforcement learning (DRL) methods, which require large training datasets, extensive tuning, and high computational resources, C-MAB learns directly from interactions with the environment. This allows it to rapidly adapt to evolving contexts (e.g., vehicle movement, traffic light transitions) without prior training or simulation. C-MAB is also advantageous over location prediction or trajectory estimation methods, which typically rely on accurate positioning data and historical mobility patterns. Instead, our approach uses high-level context information—such as proximity and direction relative to mmBS or traffic lights—making it robust even in GPS-challenged environments. However, C-MAB also has limitations. Its performance can degrade in large or continuous context spaces due to the need to estimate a reward for every context–action pair. Additionally, C-MAB does not capture long-term or delayed rewards, which could limit its performance in sequential decision tasks with temporal dependencies. To address such scenarios, future work may explore hierarchical or hybrid learning frameworks capable of modeling temporally extended behavior.

The proposed algorithm does not directly estimate or rely on channel coherence time, but rather focuses on link-level stability through the observed beam sojourn time. At mmWave frequencies, where highly directional transmissions are used, the concept of beam coherence time becomes more critical than traditional channel coherence time. Beam coherence time denotes the interval during which a beam remains aligned with a moving user before misalignment occurs and is typically an order of magnitude longer than the channel coherence time. As argued in [29], frequent realignment based on channel coherence intervals introduces excessive overhead; instead, beam realignment should occur on the timescale of the beam coherence time to maintain efficient operation. In our model, beams are fixed with a 30° beamwidth, which inherently allows for longer beam coherence times. Beam reselection is triggered only when a user exits the beam sector or when the SNR falls below a defined threshold. This design choice reduces the need for frequent physical layer realignment and is well suited for urban vehicular speeds between 30 and 50 km/h (approximately 8–13.8 m/s). Under such conditions, beam coherence times are typically on the order of seconds, allowing our algorithm sufficient temporal margin to learn and select stable beam associations. Even under higher mobility, the C-MAB framework successfully identifies long-lasting beam–IRS paths, improving robustness to short beam coherence intervals and reducing control overhead associated with frequent beam association.

### 5.1. Effectiveness of C-MAB on Connection Time

**Figure 3:**
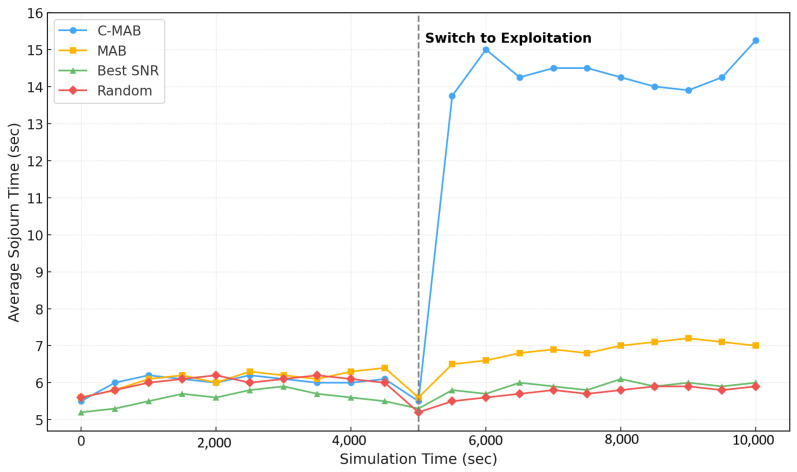
Average beam sojourn time performance of different beam selection schemes.

We conducted a comparative analysis of the C-MAB algorithm against classical MAB, Best-SNR, and random beam selection schemes, focusing on the average beam sojourn time. In the Best-SNR approach, the base station opts for a vehicle reporting the strongest received signal strength in a greedy manner. In our C-MAB, we leverage our contextual framework stated in Section 4. The beam sojourn time performance among the considered schemes is compared in Figure 3. After the exploration phase, C-MAB exhibits a notable increase, while MAB demonstrates only a slight uptick in mean beam sojourn time. This disparity is attributed to C-MAB’s proficiency in leveraging contextual information for decision-making, enabling it to outperform MAB by a factor of two, and significantly more when contrasted with Best-SNR and random selection schemes.

**Figure 4:**
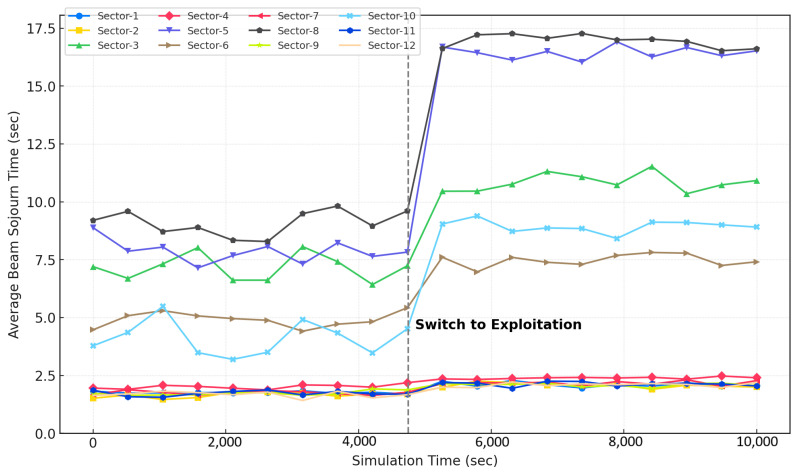
Average beam sojourn time of twelve beams with C-MAB.

In Figure 4, the beam sojourn time performance of the proposed C-MAB is illustrated for all beams. Following the exploration phase, there is a notable increase in the mean beam sojourn time for most beams as the algorithm transitions into exploration. This phenomenon is attributed to the increased utilization of certain beams in specific contexts, leading to longer beam sojourn times during the learning phase. Notably, Beam-8, Beam-5, and Beam-3, along with Beam-10 and Beam-6, exhibit enhanced mean beam sojourn times. The scenario portrayed in Figure 1 is in line with this observation, where the shaded areas for these beams predominantly represent extended road coverages due to IRSs and higher line-of-sight (LOS) instances owing to fewer obstructions.

**Figure 5:**
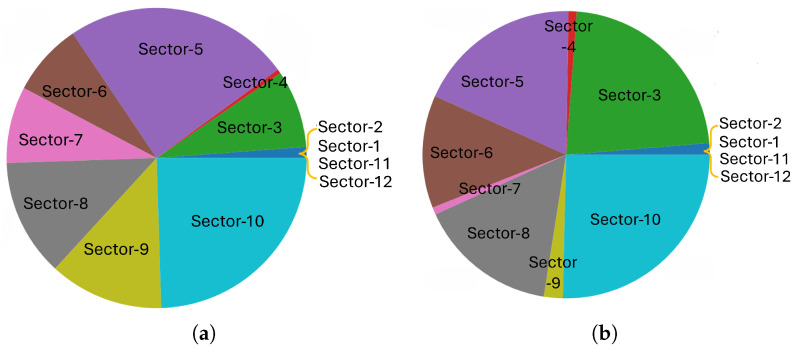
Connection frequency distribution across different beam sectors during (**a**) the exploration phase and (**b**) the exploitation phase after decision given by C-MAB algorithm.

Similar observations can be deduced from Figure 5, which illustrates the total utilization ratio of each beam throughout the exploration and exploitation phases. During the exploration phase, initial beam–sector selections are randomized across sectors. Sectors 2, 12, 11, and 1 exhibit an extremely low selection frequency due to inherently minimal vehicle traffic and shorter connection range due to weak received signal strength. Sector 4 similarly experiences limited selection since it lacks IRS deployment, compelling vehicles traveling north to primarily rely on extended coverage from neighboring Sector 3, which benefits significantly from IRS support. Sectors 7 and 9 display higher selection rates during exploration due to heavy traffic on the main road; however, their selections drastically shrink during exploitation due to short sojourn times. In contrast, Sector 3 experiences a substantial increase in connection frequency during the exploitation phase, driven by the synergistic effect of extended signal coverage from IRS deployment and prolonged connectivity opportunities resulting from vehicles frequently stopping and waiting at adjacent traffic lights. The C-MAB algorithm dynamically adjusts beam–sector selection by prioritizing sectors that offer longer and more stable connection times during the exploitation phase, hence responding effectively to contextual conditions. It intelligently considers IRS presence and infrastructure placement, consistently selecting sectors with durable, high-quality connections while systematically deprioritizing sectors characterized by transient, unstable connections. Consequently, the proposed C-MAB adeptly deal with environmental and mobility dynamics by aligning specific contexts with available beams to maximize rewards.

The C-MAB framework is computationally lightweight, making it amenable to real-time implementation at base stations or edge servers. At each decision epoch, the algorithm performs a lookup in the reward table indexed by the current context and selects the beam–IRS pair with the highest historical average. Assuming a bounded context space (e.g., 36 combinations from 3 distance bins × 3 directions × 2 traffic states), the table remains compact. No gradient updates or offline training are required. We empirically observe rapid convergence: Figure 3 shows that the proposed C-MAB outperforms the classical MAB by a factor of two only after 5000 s of exploration time. To further improve efficiency, future extensions may apply context pruning strategies that remove low-utility or rare contexts from the table, thus focusing learning on high-value decision regions.

### 5.2. The Impact of IRSs on Connection Time

The performance of C-MAB with and without IRS is also shown in Table 2. We assume that an IRS can reflect one of the beams from a BS in a 180 degree perimeter based on its orientation, which is fixed, providing coverage to one vehicle. Even though the IRS is passive and thus incurs in more path loss, when strategically placed they achieve more than a doubling in average connection time with the traditional greedy approach. This is because it is difficult for a BS to be in LoS with more than one optimum connection area, such as the waiting zone of a traffic light or a blind spot. Therefore, by using the IRS as a “mirror”, these spots become “visible” to the BS. When paired with a C-MAB algorithm, the new connection options can be more intelligently exploited, resulting in more than a quadrupling of connection time. The proposed algorithm achieves this result by “memorizing” the conditions that lead to the longest and shortest connection times. Therefore, we try to avoid connections to vehicles that find themselves in conditions that might lead to short connections and choose instead those vehicles with conditions prone to long connections.

### 5.3. The Impact of the Traffic Light Context on Connection Time

**Figure 6:**
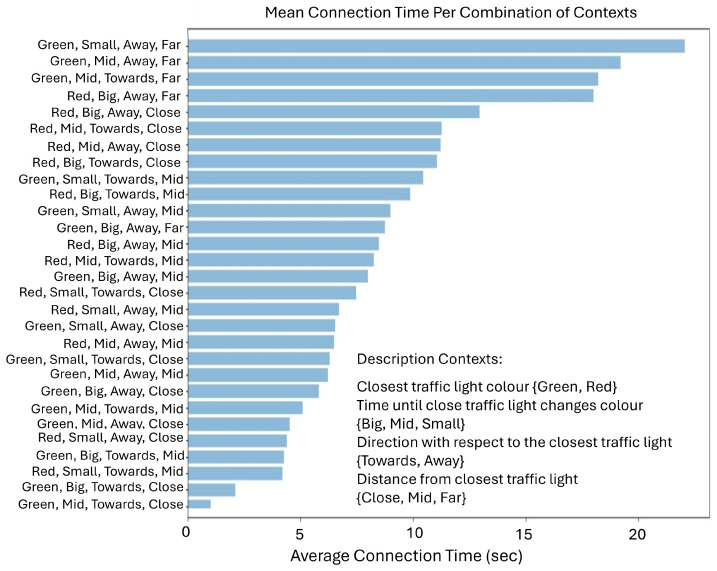
The impact of the traffic light on connection time.

Our simulation has generated various insightful examples, some of which are straightforward while others are more subtle. Generally, vehicles approaching a red traffic light tend to have longer connections compared to those approaching green lights. However, multiple context variables and numerous connection points are evaluated simultaneously, creating interactions that can amplify or diminish individual effects, thus increasing the complexity of the analysis. Figure 6 specifically evaluates the combined contexts related to traffic lights. To produce this figure, simulation data was processed by calculating the average connection duration for each combination of contexts observed. The range between the shortest and longest bars indicates the discriminatory power of these combined contexts—wider variations signify stronger predictive capabilities. For instance, the bar with the longest average connection time corresponds to a scenario labeled “Green, Small, Away, Far”, reaching approximately 24 s, although intuitively a vehicle moving away from a green light that is about to change does not have a relationship directly. This result emerges, in the considered scenario where two traffic lights are at the end main road, when a vehicle moving away from one traffic light is simultaneously approaching another traffic signal at the opposite end of the main road and eventually waits at the second light for an extended period.

Conversely, scenarios positioned at the shorter end of the chart, such as “Green, Mid, Towards, Close,” yield predictably shorter connection durations due to vehicles swiftly passing through a green light without stopping. A representative middle-range scenario, for example, the eighth-longest bar (“Red, Big, Towards, Close”), illustrates a vehicle approaching a nearby red traffic signal that will remain red for a longer time, thus extending the connection duration slightly beyond the waiting time at the light. These nuanced findings underline the value of leveraging machine learning to determine, through iterative exploration, which combinations of contexts genuinely maximize connectivity performance. Rather than relying solely on intuitive assumptions, this empirical approach ensures that subtle yet critical interactions between multiple contextual variables are effectively identified and utilized.

## 6. Conclusions

As new technologies constantly emerge to improve the future generation networks, some existing techniques in mobility management may become under-performing which directly challenge the overall network performance. Research interests in mobility management will be renewed to seek for improved solutions or novel solutions to integrate with the emerging technologies. The use of IRSs immediately challenge the beam-based mobility management by creating more complex multi-cell multi-IRS scenarios where multi-point beam switching will become a major problem and require new techniques and analytical approaches to maintain connectivity across the various network attachment points.

In scenarios with large networks high density of BSs, IRSs, and vehicles, the action space expands to include multi-beam–IRS combinations. This can be accommodated by extending the reward table and leveraging spatial grouping of IRS regions to reduce complexity. The proposed C-MAB framework is also highly scalable and remains computationally feasible under increasing vehicle density. As more vehicles enter the network, the frequency of context–beam interactions rises, which accelerates convergence and enhances learning robustness. The algorithm remains efficient due to its context-conditioned structure, which naturally partitions the learning problem. Exploring more sophisticated coordination among IRS panels, including shared context features, is part of our ongoing and future research.

## Figures and Tables

**Table 1:** Simulation parameters.

Parameter	Value
Number of beams per mmBS ($N_{b}$)	12
Carrier Frequency ($f_{c}$)	28 GHz
Transmit power	30 dBm for BS/23 dBm for vehicles
Path loss exponent (LOS/NLOS)	1.9/3.8
Noise power	−90 dBm
Vehicle Speed	30–50 km/h
Traffic light duration (Light1/Light2)	22/18 s
Simulation time	3 h
Sampling time slot	0.1 s
Simulation area	800 m × 450 m

**Table 2:** The impact of IRS usage on connection time with comparison of average connection time (in seconds) during exploration and exploitation phases with and without IRS assistance.

Case	Exploration	Explotation
With IRS	5.78	13.51
Without IRS	2.28	3.28

## Data Availability

Data is contained within the article.
